# The influence mechanism of the relationship between entrepreneurial learning and entrepreneurial intention

**DOI:** 10.3389/fpsyg.2022.1023808

**Published:** 2023-01-18

**Authors:** Cong Lin, Yan Pan, Yanli Yu, Libo Feng, Zhiyong Chen

**Affiliations:** College of Science and Technology, Ningbo University, Ningbo, China

**Keywords:** mixed-methods research, entrepreneurial learning, entrepreneurial self-efficacy, entrepreneurial intention, influence mechanism

## Abstract

Based on relevant literature, this study adopted entrepreneurial learning theory to construct a relationship model between entrepreneurial learning and entrepreneurial intention. In this framework, entrepreneurial learning was divided into three dimensions: entrepreneurial education learning, experiential learning, and social network learning. A questionnaire survey was conducted among 1,399 undergraduate students in Zhejiang Province to investigate how entrepreneurial learning influenced entrepreneurial intention, while considering the mediating effect of entrepreneurial self-efficacy. This empirical research found that: (1) experiential learning and social network learning had significant positive impacts on entrepreneurial intention, but there was no significant relationship between entrepreneurial education learning and entrepreneurial intention; (2) entrepreneurship education learning and social network learning had significant positive relationships with entrepreneurial self-efficacy, but experiential learning had a significant negative relationship with entrepreneurial self-efficacy; and (3) entrepreneurial self-efficacy partially mediated the relationship between experiential learning, social network learning, and entrepreneurial intention, and fully mediated the relationship between entrepreneurial education learning and entrepreneurial intention. These findings suggest that colleges and universities in China could broaden entrepreneurial learning and strengthen social network learning.

## 1. Introduction

China has adopted a national strategy focused on mass entrepreneurship and innovation, thus catalyzing waves of efforts that led to robust conditions for the development of both areas. The Global Entrepreneurship Watch 2018/2019 China Report indicates that although China’s current entrepreneurial activity index is high, the national success rate is relatively low when compared with the rates found in developed European countries and the United States. Furthermore, the rate of opportunity-driven entrepreneurship and innovation remains low. Since China is now in a period of economic transition, there is the need to improve these rates, and college students with higher education could be those that bear this responsibility.

Under social cognitive theory, entrepreneurship is considered as a conscious planning behavior guided by behavioral intention (Krueger et al., 2000), and many scholars show that entrepreneurial intention is the best predictor of entrepreneurial behavior (Krueger and Carsrud, 1993; Liñán et al., 2005). In earlier studies, most researchers attempted to clarify the factors that influenced entrepreneurial intentions among college students by either focusing on their demographic factors (e.g., gender, education, major, and parent occupations) or adopting a psychological perspective centered on personality traits. Although these analyses have produced valuable findings, they have not revealed a good solution for actually improving entrepreneurial abilities or behaviors in college students, making the impact of these studies not high.

Meanwhile, current entrepreneurship research focuses on both multidisciplinary and multilevel examinations, having gradually shifted from static to dynamic approaches. While scholars once had different opinions on whether entrepreneurship could be taught, most now agree that it can be. For example, Drucker (1985) emphasized that entrepreneurship was not a mysterious concept and could be learned like other disciplines, and Anselm (1993) showed that entrepreneurial learning could enhance both entrepreneurial intention and activity levels. Through entrepreneurship learning, students gain related knowledge, experience, understanding of the theory–reality gap, and skills in identifying entrepreneurial opportunities, thus improving their entrepreneurial intention (Ramsgaard and Østergaard, 2018). For college students, the outcome of entrepreneurship learning is acquiring relevant knowledge useful for enterprise creation.

The emergence of recent research on entrepreneurship learning—which make it their task to explain the formation and development of entrepreneurial self-confidence (Rae and Carswell, 2001; Ni and Wang, 2005), which is enhanced through entrepreneurial learning—reflects the current dynamic trends in entrepreneurship theory research. In an empirical study on the factors influencing entrepreneurial intention, Chen et al. (1998) posited the concept of entrepreneurial self-efficacy by combining self-efficacy theory and entrepreneurial process theory, and demonstrated that entrepreneurial self-efficacy has a positive effect on entrepreneurial intention. This implies that effective entrepreneurial learning can help college students acquire entrepreneurial knowledge and improve their entrepreneurial self-efficacy, which are both important to enhance their entrepreneurial intention.

Nevertheless, related literature on entrepreneurial learning and intention have primarily focused on the impact of individual types of entrepreneurial learning on entrepreneurial intention, such as the impacts of entrepreneurship education learning, social network learning, and experiential learning on entrepreneurship intention. Although each of these types of entrepreneurial learning were capable of explaining the impact mechanism of entrepreneurial intention, the explanatory power of these variables were not comprehensive enough. Therefore, this study places entrepreneurship education, experience, and social network learning into the framework of entrepreneurship learning, examines the impact of entrepreneurial intention, and introduces entrepreneurial self-efficacy as an intermediary variable into the research framework; this approach is novel and characterizes an improvement compared with previous research methods used to examine entrepreneurial intention.

## 2. Literature review

### 2.1. Entrepreneurial learning

Entrepreneurial learning is a dynamic process that plays an important role in entrepreneurship, making entrepreneurial learning and behavior closely related (Rae and Carswell, 2000). As entrepreneurship is a continuous learning process, relevant research often use the support of learning theory (Minniti and Bygrave, 2001). The theory of entrepreneurial learning was first explored from the perspective of economics, and it holds that entrepreneurial learning refers to creative learning that produces innovation and improves opportunity alertness (Schumpeter, 1934; Kirzner, 1973). With the continuous development of entrepreneurial learning research, scholars have made use of social cognitive theory, social learning theory, and experiential learning theory to create a framework for entrepreneurial learning theory. These developments also led scholars to be able to show that many forms of entrepreneurial learning exist, including cognitive, experiential, and observational learning (Kolb, 1984; Lumpkin and Lichtenstein, 2005; Holcomb et al., 2009). Minniti and Bygrave (2001) and Politis (2005) pointed out that entrepreneurs update their entrepreneurial knowledge through experience accumulation, which then enhances their entrepreneurial beliefs and enriches entrepreneurial learning theory.

Based on experiential learning theory, Cope (2005) argued that entrepreneurship is a dynamic learning process through which relevant knowledge and skills are acquired, with integral elements including awareness, feedback, relevance, and application. Meanwhile, Weick (1995) divided entrepreneurial learning into cognitive learning and experiential learning. Politis (2005) defined experiential learning as a process in which entrepreneurs transform direct experience from previous work into knowledge. Holcomb et al. (2009) thought that experiential learning is a process in which individuals continuously adjust and improve their own cognitive structures through intuitive inferences based on both direct and indirect experiences (observing or imitating others’ behaviors) with uncertain environments, leading to new knowledge acquirement. Working under social learning theory, Rae and Carswell (2001) proposed a theoretical model for entrepreneurial learning, wherein entrepreneurs learn entrepreneurial knowledge and gain work experience through formal systematic learning at school, experiential learning at work, and social network learning.

Therefore, in the process of entrepreneurial learning, individuals identify entrepreneurial opportunities while enhancing self-confidence and self-efficacy, thereby generating ideas for building new enterprises. Aldrich (1999) further pointed out that social networks can provide entrepreneurs with valuable entrepreneurial information, facilitate their ability to access entrepreneurial resources, and help them create new enterprises. Similarly, Kim and Miner (2007) pointed out that entrepreneurs acquire much of their knowledge about entrepreneurship from others. These remarks are especially relevant for the Chinese context because of the strong influence of traditional Confucian culture, which focuses on the benefits of social relations to groups or individuals (Tsui and Farh, 1997). That is, China’s well-established and intimate social network provide a good learning platform for entrepreneurs to acquire significant hidden assets. Given the nation’s ongoing economic transformation, social network learning should have a clear and positive impact on entrepreneurial acquisition.

### 2.2. Entrepreneurial self-efficacy

Self-efficacy theory is the foundation of entrepreneurial self-efficacy theory and a core concept in sociology theory (Bandura, 1977); some fields have described self-efficacy as being not a static characteristic but rather an alterable one, and being a reflection of an individual’s belief in own ability to perform and partake in a desired behavior. For example, based on social cognition theory, self-efficacy is generally considered to be the subjective confidence that an individual has in own ability to mobilize resources to successfully perform a behavior (Wood and Bandura, 1989). Entrepreneurial self-efficacy was first proposed by Chen et al. (1998) through a combination of self-efficacy theory with entrepreneurship research, describing that it serves to assess an individual’s confidence in own ability to run a business, including its innovation, marketing, management, financial management, and risk-taking endeavors (Chen et al., 1998). Several studies have demonstrated a positive correlation between entrepreneurial self-efficacy and entrepreneurial intention (Krueger and Carsrud, 1993; Boyd and Vozikis, 1994; Chen et al., 1998; Wilson et al., 2007), showcasing that the literature depicts entrepreneurial self-efficacy as playing an extremely important role in the process of starting a new business and being simultaneously affected by many factors, such as social network and experiential learning and the learning style of entrepreneurship education.

### 2.3. Entrepreneurial intention

Entrepreneurial intention is a career decision-making process that describes individuals’ intention to start a new enterprise and beliefs that they can take relevant practical actions in the future (Katz, 1992; Thompson, 2009; Bullough et al., 2014). It is a pre-test indicator of entrepreneurial behavior as only individuals with a certain degree of entrepreneurial intention will develop such behavior. Most empirical investigations on entrepreneurial intention have primarily based themselves on the theory of planned behavior, entrepreneurial event theory, and entrepreneurial self-efficacy theory. In this study, we set entrepreneurial self-efficacy as an intermediary variable and used entrepreneurial self-efficacy theory to explore the influence mechanism of entrepreneurial learning on entrepreneurial intention.

Entrepreneurial self-efficacy has been shown to have a significant positive effect on entrepreneurial intention (Bird, 1988), and Boyd and Vozikis (1994) later revised Bird’s entrepreneurial intention model by introducing self-efficacy. Thus, entrepreneurial self-efficacy can enhance self-confidence on own entrepreneurial ability, potentially producing a strong entrepreneurial intention. This study further theorizes about the impact mechanism of entrepreneurial self-efficacy and entrepreneurial learning on entrepreneurial intention, thus providing more theoretical resources for researchers to use in future empirical research on entrepreneurial intention.

## 3. Research design

In this study, we used a mixed-methods research design to comprehensively examine the entrepreneurial learning methods that can enhance college students’ entrepreneurial intention. Mixed-methods research combines the advantages of both qualitative and quantitative methods: the first enable researchers to explore target phenomena, identify their key factors, and develop a deep understanding of theoretical model construction, while the latter provides a more pragmatic way to test relationships between proposed theories and a hypotheses. Thus, in Study 1, we conducted qualitative interviews to identify the major entrepreneurial learning styles influencing entrepreneurial intention. In Study 2, we established the research model by integrating the findings of Study 1 on the entrepreneurial learning styles that influence entrepreneurial intention and then verified the validity of this model using a questionnaire survey. The research question was: which entrepreneurial learning styles affect entrepreneurial intention?

## 4. Study 1: Qualitative research

### 4.1. Methods

The interviews with college entrepreneurs were conducted from January 2022 to February 2022, and the maximum interview time was set to 30 min. The interviews aimed to identify the main impact of entrepreneurial learning style on entrepreneurial intention. The interview procedures were performed based on the usual processes of interview content analysis (French et al., 2017). Recruitment was conducted using convenience and snowball sampling methods, with participants being familiar college entrepreneurs who gave references of their contacts for interviews. Snowball sampling was used to recruit the most relevant interviewees through the contact information provided by other stakeholders (Noy, 2008). Monetary incentives were offered to encourage suggestions from qualified interviewees. Respondents were asked to briefly describe their entrepreneurial learning experience while focusing on entrepreneurial learning, intention, and the entrepreneurial learning methods that affect intention, which then led to open questions. Interview contents were recorded in detail. At the end of the interview, interviewees were asked to check the interview records and confirm their accuracy. In the discussions below, we used the interviewees’ last name in order to ensure anonymity. Supplementary Table 1 provides information on participants’ sociodemographic characteristics.

Two independent evaluators examined the content of the interviews and identified entrepreneurial learning styles that influenced entrepreneurial intention. After completing the coding process, the evaluators compared their independent coding results to determine and discuss instances of coding match and mismatch. When the evaluators could not agree on a code, the code was not included in the classification. An encoder reliability score of 92% is deemed as acceptable in prior research (Lombard et al., 2002). Ultimately, the independent evaluators could reach consensus, resulting in a series of key factors that were considered to affect entrepreneurial intention (Table 1).

**TABLE 1:** Factors for participating in EI.

Aspect	Time mentioned	# of people
Entrepreneurship education learning	19	15
Social network learning	15	14
Experiential learning	13	12
Cognitive learning style	1	1
Learning ability	1	1
Learning skills	1	1

### 4.2. Results

The first factor identified was entrepreneurship education learning, comprising descriptions about the following: creative entrepreneurship atmosphere on campus; social and management abilities cultivated by the curriculum; entrepreneurship knowledge acquired through the curriculum; the establishment and support of entrepreneurship associations. Some quotations that exemplify this factor are described herein: “In school, I learned some entrepreneurship courses and participated in the Internet entrepreneurship competition, which sprouted the idea of entrepreneurship in me” (Wang), “When I was in charge of the entrepreneurship practice project in the university, I improved my management ability and played an important role in shaping the future entrepreneurship project” (Zhang), “The school attaches great importance to the Internet entrepreneurship competition and has a relatively strong entrepreneurial atmosphere, which helps stimulate our entrepreneurial ideas” (Lin), “The school’s entrepreneurial community played a good role in the formation of the entrepreneurial team” (Chen).

The second factor identified was social network learning, comprising descriptions about the following topics: exchange of entrepreneurial experience and knowledge between relatives and friends; contact and exchange involving government personnel; involving professional investment institutions; involving potential customers and suppliers. Herein we exemplify the second factor with some quotations from participants: “My father has his own business, and his exchanges make me think of my own plan to open a company” (Li), “The exchanges with the staff of the Human Resources Security Bureau of the government enables me to understand preferential policies to support college students’ entrepreneurship, which may help me start a business” (Wu).

The third factor identified was experiential learning, comprising descriptions on the following matters: Experiences accumulated in the process of starting a business; decisions made based on experience; lessons learned from failure. Some examples of quotations related to experiential learning are described herein: “I encountered a lot of difficulty in the process, and my ability to continue was acquired through the experience I accumulated” (Xu), “I have worked in a foreign trade company for 2 years. During this period, I have accumulated a lot of sales experience, which inspired me to start my own business” (Sun).

All factors listed by individual respondents were considered insignificant and were not used for further analysis. The excluded factors included cognitive learning style, learning ability, and learning skills.

### 4.3. Discussion

The three factors identified in Study 1 contributed to a deeper understanding of the determinants of entrepreneurial intention among college students, which were then applied to Study 2’s research model. Then, we quantitatively examined the impact of these factors on entrepreneurial intention.

## 5. Theoretical model

**FIGURE 1:**
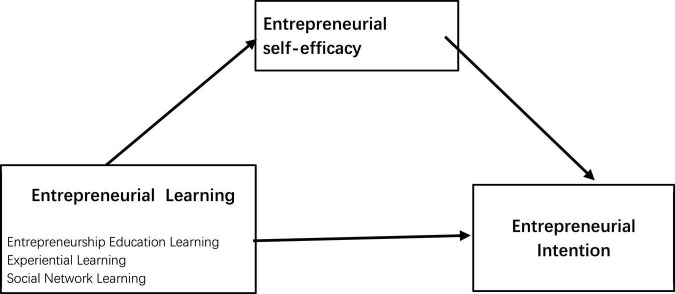
Theoretical model.

This study adopted entrepreneurial learning theory and conducted path analysis to examine how entrepreneurial learning influences entrepreneurial intention while considering entrepreneurial self-efficacy as an intermediary variable. Figure 1 illustrates the theoretical model of the influence mechanism by which entrepreneurial learning impacts entrepreneurial intention.

### 5.1. Research hypotheses

#### 5.1.1. The relationship between entrepreneurial learning and entrepreneurial intention

According to Petkova (2009), entrepreneurs set goals when facing new tasks and environments through entrepreneurial learning, which then stimulate possible behaviors. The role of entrepreneurial learning can also be assessed from the perspective of entrepreneurship education, which directly impacts entrepreneurial intention (Souitaris et al., 2007; Mason and Arshed, 2013; Lv et al., 2021). Furthermore, Aliedan et al. (2022) pointed out that entrepreneurship education plays a key role in the cultivation of entrepreneurial intention. Gwynne (2008) describes that entrepreneurship education directly affects and is a key tool for enhancing entrepreneurial intention. Fragoso et al. (2020) conducted a study with Brazilian and Portuguese university students and confirmed that entrepreneurship education and training significantly positively impact entrepreneurial intention. For entrepreneurs, the combination of schooling in the early stages of their career and previous work experience can increase the knowledge needed to manage new ventures and stimulate their intention to start a business (Rae and Carswell, 2001). Additionally, Fayolle et al. (2006) showcased that entrepreneurship education has a significant positive impact on college students’ entrepreneurial intention. Two other studies further confirmed that entrepreneurship education effectively improves entrepreneurial intention, and described that the latter is the best index for predicting individual entrepreneurial behavior (Dehghanpour Farashah, 2013; Zhang et al., 2014). Moreover, some scholars have found that entrepreneurship education improves entrepreneurial alertness by cultivating college students’ creativity, in turn having a significant impact on their entrepreneurial intention (Obschonka et al., 2017). Thus, entrepreneurial learning creates a good entrepreneurial atmosphere and has a positive impact on entrepreneurial intention.

Sequeira et al. (2007) proposed a formation model for initial entrepreneurial behavior wherein social network learning is mainly accomplished by observing and learning from successful entrepreneurs within the network; in this model, social persuasion from network members can induce the belief that individuals can achieve entrepreneurship, which influences their entrepreneurial intention. Furthermore, Scherer et al. (1989) reported that between 35 and 70% of entrepreneurs are influenced by successful entrepreneurs within their social network, and that they continuously gain entrepreneurial experience and knowledge through this network, thereafter further stimulating their intention. BarNir et al. (2011) believes that social networks also promote role model learning, which not only provides valuable information about entrepreneurial opportunities but also sound entrepreneurial process guidance services and strong emotional support, thus enhancing individual entrepreneurial intention. According to Hills et al. (1997), the entrepreneurial opportunities identified by entrepreneurs who use social networks to mine entrepreneurial resources are significantly better than those identified by entrepreneurs who use solely the work environment. Overall, social networks can encourage and support entrepreneurial activity (Aldrich and Zimmer, 1985).

In addition, when examining entrepreneurial intentions among college students, several studies have shown the following results: significant and positive effects of encouragement and support from family members, relatives, and friends (Davidsson and Honig, 2003; Baughn et al., 2006); practical entrepreneurship education organization can positively affect the entrepreneurship learning process and increase entrepreneurship alertness and intention (Kuckertz, 2013); Luo et al. (2022) revealed that social relations can significantly impact and promote the entrepreneurial intention of college students; practical entrepreneurial learning helps students acquire relevant entrepreneurship experience, which both increases their understanding of the theory–reality gap and ability to identify entrepreneurial opportunities, thereby improving entrepreneurial intention (Ramsgaard and Østergaard, 2018).

According to these studies, scholars have categorized entrepreneurial learning styles based on different research perspectives, with a focus on college students’ conditions. Meanwhile, this study analyzed how entrepreneurial learning impacted intention by dividing the first into three components, namely entrepreneurial education, experiential, and social network learning. This led to the following hypotheses:

H1: Entrepreneurial education learning has a significant positive relationship with entrepreneurial intention.

H2: Experiential learning has a significant positive relationship with entrepreneurial intention.

H3: Social network learning has a significant positive relationship with entrepreneurship.

#### 5.1.2. The relationship between entrepreneurial learning and entrepreneurial self-efficacy

Studies show that entrepreneurial learning can enhance self-confidence and promote behavioral processes relevant to the entrepreneur’s knowledge set. Specifically, entrepreneurial learning can improve entrepreneurial self-efficacy (Minniti and Bygrave, 2001), and Saeed et al. (2015) described that perceived educational support exerted the highest influence on entrepreneurial self-efficacy. Taking a qualitative approach, Rae and Carswell (2001) constructed a model of entrepreneurial learning, which is described as being able to improve entrepreneurial self-confidence and self-efficacy. Zhao et al. (2005) conducted two longitudinal surveys at two periods with 265 MBA students from five American universities, and then tested the relationships between entrepreneurship education learning, self-efficacy, and intention through a structural equation model; their results showed that entrepreneurship learning had a significant positive impact on entrepreneurial self-efficacy. Entrepreneurship education has also been shown to influence individuals to develop business plans and participate in sand table exercises, thereby improving their entrepreneurial self-efficacy (Wilson et al., 2007). Furthermore, entrepreneurs directly acquire achievement experience and foster a strong entrepreneurial self-efficacy through the repeated acquisition of outstanding achievements (Bandura, 1977). Thus, entrepreneurs can enhance their entrepreneurial self-efficacy not only through direct experience but also observational learning that helps them gain such experience.

Finally, Herron and Sapienza (1992) showed that the skills acquired through past achievements strengthen individual self-efficacy and promote the achievement of higher expectations, and a survey conducted with 804 college students in Zhejiang Province revealed that entrepreneurship education has a positive impact on entrepreneurial self-efficacy (Wu et al., 2021). These descriptions led to the following hypotheses:

H4: Entrepreneurship education learning has a significant positive relationship with entrepreneurial self-efficacy.

H5: Experiential learning has a significant positive relationship with entrepreneurial self-efficacy.

H6: Social network learning has a significant positive relationship with entrepreneurial self-efficacy.

#### 5.1.3. The relationship between entrepreneurial self-efficacy and entrepreneurial intention

Entrepreneurial self-efficacy is a pre-test variable and an important predictor of entrepreneurial intention and behavior, as well as plays an active role in the formation and development of entrepreneurial intention (Boyd and Vozikis, 1994). Sequeira et al. (2007) found that high entrepreneurial self-efficacy and a strong social network enhanced individual entrepreneurial intention. Based on a comparison of two samples of American and Korean business school students and full-time managers, De Noble et al. (1999) found that entrepreneurial self-efficacy had a positive effect on entrepreneurial intention. Bullough et al. (2014) conducted a study with 272 entrepreneurs in Afghanistan, finding that high entrepreneurial self-efficacy could develop entrepreneurial intentions, even in a war environment.

Research also showed that individual entrepreneurial self-efficacy can be provided through education and training, thereby increasing the rate of potential entrepreneurial activity (Florin et al., 2007). Additionally, past experience and behavior can enhance entrepreneurial self-efficacy and, subsequently, the entrepreneurial intention of future entrepreneurs (McGee and Peterson, 2019). Zhao et al. (2005) conducted a survey with the same group of MBA students at an interval of 2 years, confirming that entrepreneurial self-efficacy was strongly and positively correlated with entrepreneurial intention. Within the context of Chinese college students, numerous scholars have carried out empirical research *via* questionnaire surveys and confirmed the significant positive impact of entrepreneurial self-efficacy on entrepreneurial intention (Liu et al., 2019; Chien-Chi et al., 2020; Li et al., 2020). Thus, the following hypothesis was developed:

H7: Entrepreneurial self-efficacy is significantly related to entrepreneurial intention.

#### 5.1.4. The mediating role of entrepreneurial self-efficacy

Entrepreneurial self-efficacy was shown to fully mediate the relation between entrepreneurship-related curriculum learning and entrepreneurial intention (Chou et al., 2011). Yang et al. (2021) conducted a questionnaire survey with 1,393 college students across six universities, then conducted an empirical analysis to investigate how the entrepreneurship curriculum affected their entrepreneurial intention. The results showed that entrepreneurial self-efficacy played a particularly important intermediary role. Furthermore, many scholars have confirmed that entrepreneurial self-efficacy plays an intermediary role in the relationships between entrepreneurial education, the social network, environmental support, and many other factors related to entrepreneurial intentions among college students (Alam et al., 2015; Jiang et al., 2017; Roy et al., 2017). Meanwhile, entrepreneurial learning increases individual self-efficacy, which enhances individual entrepreneurial intention (Zhao et al., 2005; Wilson et al., 2007). In addition, Jiatong et al. (2021) conducted a survey with Chinese business students and found that entrepreneurial self-efficacy played a partial mediating role in the association of entrepreneurship education and entrepreneurial intention. This finding was supported by the results of the study by Li et al. (2020), who examined Chinese American college students’ entrepreneurial behavior. Based on the above, the following hypotheses were developed:

H8: Entrepreneurial self-efficacy plays a mediating role in the relationship between entrepreneurial education learning and entrepreneurial intention.

H9: Entrepreneurial self-efficacy plays a mediating role in the relationship between experiential learning and entrepreneurial intention.

H10: Entrepreneurial self-efficacy plays a mediating role in the relationship between social network learning and entrepreneurship intention.

## 6. Study 2: Quantitative research

### 6.1. Research approach

This study collected data through a questionnaire survey. The questionnaire content was developed based on Study 1’s data and by referencing the questionnaire design in previous literature, and the survey was conducted between March and May 2022. At present, there are 37 public universities and 23 private universities in Zhejiang Province; thus, 12 universities, including 7 public and 5 private universities, were randomly sampled.

To ensure sample quality, three indicators were used to screen the responses. First, according to an initial pretest’s completion time, it took 5 to 10 min to complete the questionnaire. Therefore, based on prior research, participants finishing the survey within 3 min were regarded as not filling it in responsibly, excluding their questionnaire from analysis. Second, a reverse question was included in the questionnaire design, so if a student gave a non-reversed answer, the questionnaire was excluded. Finally, questionnaires with repetitive results or extreme values were excluded. Participants were recruited through convenience sampling to reduce deviation, and the online survey website platform Questionnaire Star was used for survey dissemination. To improve recovery rate and effectiveness, teachers who were responsible for student work were selected to distribute and collect the questionnaires. Finally, 1,500 questionnaires were collected, of which 1,399 were valid (recovery rate of 93.3%).

The basic sociodemographic data of participants is as follows: 599 were male students (42.8%) and 800 were female students (57.2%); 366 students were in grade one (26.2%), 372 in grade two (26.6%), 336 in grade three (24%), 293 in grade four (20.9%), 32 in grade five (2.3%); 403 were science and engineering majors (28.8%), 387 economics and management majors (27.7%), 300 humanities, law and philosophy, and society majors (21.4%), 258 art and design majors (18.4%), and 51 other majors (3.6%). Moreover, 286 students participated in entrepreneurship competitions (20.4%) and 1,113 did not (79.6%). As for parental factors, 638 had parents with entrepreneurial experience (45.6%), while 761 had parents with no entrepreneurial experience (54.4%).

### 6.2. Research tools

The main questionnaire variables were entrepreneurial learning (i.e., entrepreneurial education, experiential, and social network learning), entrepreneurial self-efficacy, and entrepreneurial intention. All questionnaire items were rated using a 5-point Likert scale. SPSS version 22 and AMOS version 24 were used to analyze variable relationships.

#### 6.2.1. Entrepreneurial learning

For the entrepreneurship learning scale, we mostly used scales developed by Hansen (1995), Franke and Lüthje (2004), Corbett (2007), and other scholars, while combining the maturity scale compiled in the specific context of our country, as divided into three dimensions (i.e., entrepreneurship education, experiential, and social network learning). Entrepreneurship education learning comprised four topics: creative entrepreneurship atmosphere on campus, social and management abilities cultivated by the curriculum, entrepreneurship knowledge acquired through curriculum, and the establishment and support of entrepreneurship associations. Experiential learning consisted of three parts: the experience accumulated in the process of starting a business, the decisions made based on experience, and lessons learned from failure. Social network learning comprised four topics: exchange of entrepreneurial experience and knowledge between relatives and friends, and contact and exchange involving government personnel, contact and exchange involving professional investment institutions, and contact and exchange involving potential customers and suppliers.

#### 6.2.2. Entrepreneurial self-efficacy

The entrepreneurial self-efficacy scale mainly drew upon the scales of Liñán and Chen (2009) and other scholars, and it comprised three items: self-confidence to start a company, the ability to control the whole process of entrepreneurial self-confidence, and ability to clear the details of starting a business.

#### 6.2.3. Entrepreneurial intention

The entrepreneurial intention scale mainly drew upon scales from Zhao et al. (2005), van Gelderen et al. (2008), and other scholars. The five items were: “I think I will start a business in the future,” “I will go all out to start my own business,” “Starting my own company is my real interest,” “I have systematic and in-depth thoughts about running my own company,” and “I have made full preparations for starting my own company.”

Under the constructed theoretical model, the first-order confirmatory factor analysis showed that the factor loadings of each item should ideally be higher than 0.7, and reliability was poor for items with factor loadings lower than 0.7. Thus, the corresponding items were deleted to ensure overall reliability. Specifically, the item on “the establishment and support of entrepreneurship associations” was deleted from the entrepreneurship education learning subscale, which ultimately consisted of three items. Furthermore, one question (“I have systematic and in-depth thoughts about running my own company“) was deleted from the entrepreneurship intention scale, which ultimately consisted of four items. Meanwhile, other items met the requirements of the relevant structural equation indicators, such as the fitting degree.

### 6.3. Results

#### 6.3.1. Component reliability and convergent validity

According to Jackson et al. (2009), the measurement model should be evaluated prior to the structural equation analysis, such that the former can evaluate the latter. The main indexes for evaluating measurement models include composite reliability (CR) and average variance extracted (AVE). CR values refer to the combined reliability of all measurement variables, thus indicating the internal consistency of the structure index, with higher CR values indicating higher internal consistency. In this study, a CR of 0.7 was considered as an acceptable threshold (Fornell and Larcker, 1981). Meanwhile, AVE is the average of the explanatory power of the latent variables to the measured variables, and higher AVEs indicate higher convergent validity for the given dimension. In this study, the AVE should be greater than 0.5, with a value between 0.36–0.5 being considered an acceptable threshold (Fornell and Larcker, 1981).

This study imported survey data from all 1,399 participants into AMOS, then conducted a confirmatory factor analysis on the measurement model constructed for each dimension. As such, CR and AVE values were obtained (Table 2). Table 2 shows that the CR for the five dimensions exceeded 0.8, the AVE value exceeded 0.6, and the *p*-value was significant (<0.001), that is, the measurement model showed good composition reliability and convergent validity.

**TABLE 2:** Validation factor analysis for each dimension.

Construct	Item	Unstd	S.E.	*z*-value	*P*	Label	Std	SMC	CR	AVE
EI	I think I will start a business in the future	1.000					0.875	0.766	0.910	0.716
	I will go all out to start my own business	1.020	0.023	43.986	***		0.888	0.789		
	Starting my own company is my real interest	0.951	0.026	37.150	***		0.800	0.640		
	I have made full preparations for starting my own company	0.992	0.026	38.549	***		0.818	0.669		
EEL	Creative entrepreneurship atmosphere on campus	1.000					0.825	0.681	0.873	0.696
	Social and management abilities cultivated by curriculum learning	0.951	0.028	33.998	***		0.872	0.760		
	Entrepreneurship knowledge acquired through the curriculum	0.917	0.028	32.394	***		0.804	0.646		
EL	The experience accumulated in the process of starting a business	1.000					0.873	0.762	0.898	0.745
	The decisions made based on experience	0.956	0.024	39.808	***		0.870	0.757		
	Lessons learned from failure	0.958	0.025	38.683	***		0.846	0.716		
SNL	Exchange of entrepreneurial experience and knowledge between relatives and friends	1.000					0.848	0.719	0.926	0.757
	Contact and exchange involving government personnel	1.025	0.025	41.446	***		0.869	0.755		
	Contact and exchange involving professional investment institutions	1.077	0.024	44.102	***		0.903	0.815		
	Contact and exchange involving potential customers and suppliers	1.043	0.026	40.689	***		0.859	0.738		
ESE	Self-confidence to start a company	1.000					0.891	0.794	0.915	0.782
	The ability to control the whole process of entrepreneurial self-confidence	1.014	0.022	46.072	***		0.904	0.817		
	Ability to clear the details of starting a business	0.988	0.023	42.890	***		0.857	0.734		

***p < 0.001. EI, entrepreneurial intention; EEL, entrepreneurial education learning; EL, experiential learning; ESE, entrepreneurial self-efficacy; SNL, social network learning.

#### 6.3.2. Discriminant validity

Discriminant validity serves to assess whether items which measure one dimension have high loadings on other dimensions or whether items exhibit a cross-loading phenomenon (Bagozzi and Yi, 2012; Nunkoo et al., 2013). Table 3 shows the results for the analyses on discriminant validity, wherein the root value of AVE was greater than the correlation value between other dimensions; thus, each scale showed good discriminant validity.

**TABLE 3:** Discriminant validity.

	Convergence validity		Discriminant validity			
	AVE	EI	ESE	SNL	EL	EEL
EI	0.910	**0.954**				
ESE	0.915	0.648	**0.957**			
SNL	0.926	0.614	0.797	**0.962**		
EL	0.898	0.439	0.257	0.338	**0.948**	
EEL	0.873	0.484	0.467	0.508	0.643	**0.934**

The bold diagonal shows the root of the AVE value, while the lower triangle is the pearson correlation value. EI, entrepreneurial intention; EEL, entrepreneurial education learning; EL, experiential learning; ESE, entrepreneurial self-efficacy; SNL, social network learning.

#### 6.3.3. Structural equation modeling and analysis

**FIGURE 2:**
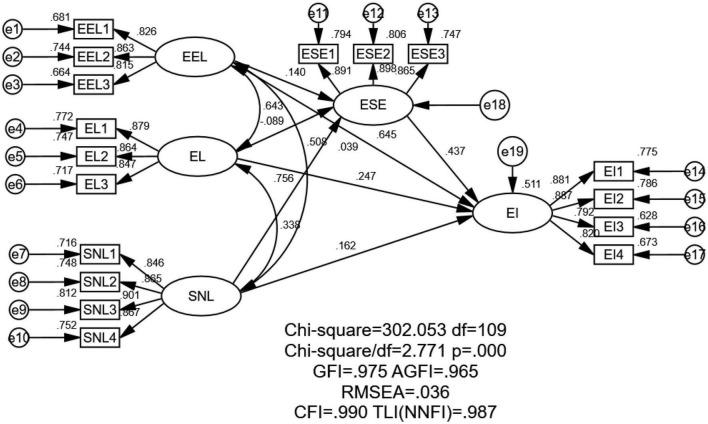
Structural equation model results. EI, entrepreneurial intention; EEL, entrepreneurial education learning; EL, experiential learning; ESE, entrepreneurial self-efficacy; SNL, social network learning.

According to the results for composition reliability, average variance extraction quantity, and discriminant validity of the five dimensions, the structural equation model was suitable. As shown in Figure 2, we referred to the relevant literature to construct a structural equation model that could explore the impact mechanism of the relationship between entrepreneurial learning and entrepreneurial intention.

The goodness-of-fit correlation indexes of that model, as analyzed by analyzed by AMOS version 24, were as follows: Chi-squared/df = 2.771, which was less than 3; GFI = 0.975, AGFI = 0.965, CFI = 0.990, TLI = 0.987, which were all more than 0.9; RMSEA = 0.036, which was less than 0.08; SRMR = 0.0217, which was less than 0.05. When the sample size is more than 200, the significance of the model can easily reach the significant level without marking. These fit indexes showed that the model fitted the data very well.

#### 6.3.4. Model global structure test

As shown in Table 4, the regression coefficients and parameter estimates of the latent variables obtained *via* AMOS 24 show the following: the significant *P*-value of experiential learning to entrepreneurial self-efficacy was 0.002 (i.e., less than 0.01), the significant *P*-value of entrepreneurial education learning was 0.27 (i.e., more than 0.05), and the significant *P*-values of other dimensions were less than 0.001. The results show that entrepreneurial self-efficacy had a significant impact on entrepreneurial intention, while entrepreneurial education learning and social network learning had significant positive impacts on entrepreneurial self-efficacy, and experiential learning had a significant negative impact on entrepreneurial self-efficacy. There were no significant effects between entrepreneurial education learning and entrepreneurial intention, experiential learning, and social network learning, while entrepreneurial self-efficacy had a significant positive effect on entrepreneurial intention. These findings support H2, H3, H4, H6, and H7, but reject H1 and H5, although the two dimensions in H5 had negative and significant effects.

**TABLE 4:** Estimation of regression coefficient and parameter.

			Estimate	S.E.	C.R.	*P*
ESE	<−−−	EEL	0.149	0.034	4.44	***
ESE	<−−−	EL	−0.087	0.028	−3.166	0.002
ESE	<−−−	SNL	0.792	0.029	27.044	***
EI	<−−−	EEL	0.042	0.038	1.103	0.27
EI	<−−−	ESE	0.444	0.043	10.363	***
EI	<−−−	EL	0.247	0.032	7.791	***
EI	<−−−	SNL	0.173	0.045	3.84	***

***p < 0.001. EI, entrepreneurial intention; EEL, entrepreneurial education learning; EL, experiential learning; ESE, entrepreneurial self-efficacy; SNL, social network learning.

As shown in Table 4, entrepreneurial self-efficacy fully mediated the relationship between entrepreneurial education learning and entrepreneurial intention; partially mediated the relationship between experiential learning and entrepreneurial intention; partially mediated the relationship between social network learning and entrepreneurial intention. These results support H8, H9, and H10.

## 7. Discussions and conclusion

### 7.1. Research discussions

**FIGURE 3:**
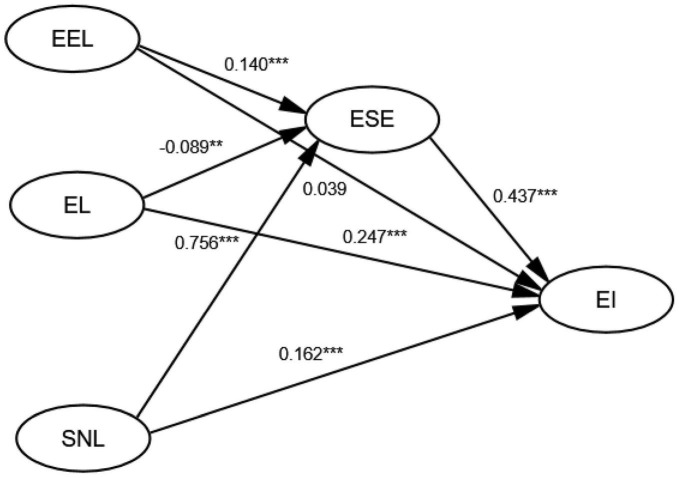
Structural equation model results: path diagram. EI, entrepreneurial intention; EEL, entrepreneurial education learning; EL, experiential learning; ESE, entrepreneurial self-efficacy; SNL, social network learning. ^**^p < 0.01 and ^***^p < 0.001.

This study conducted an empirical analysis that led to the following conclusions (Figure 3): Entrepreneurship education learning had a significant positive impact on entrepreneurial self-efficacy, in line with the findings of the studies by Gwynne (2008), Dehghanpour Farashah (2013), Zhang et al. (2014), and Obschonka et al. (2017). Social network learning was significantly positively correlated with entrepreneurial self-efficacy, and the path coefficient between them was 0.756, the largest influence coefficient among all latent variables. Therefore, entrepreneurial self-efficacy plays a crucial intermediary role in the association of social network learning and entrepreneurial intention.

Due to Confucianism, people in China attach great importance to interpersonal relationships, making it so that the suggestions of parents and friends can impact college students’ entrepreneurship-related decisions. Furthermore, China’s long-term and close social networks provide a good learning platform where entrepreneurs can obtain significant entrepreneurial resources. Based on the path coefficients in the structural equation model, our results show that social network learning had the greatest impact on enhancing entrepreneurial self-efficacy in our sample. However, experiential learning had a significant negative effect on entrepreneurial self-efficacy, which is contrary to the results of previous literature (Bandura, 1977; Wilson et al., 2007; Holcomb et al., 2009), and constitutes a new finding of this study. This outcome could be related to the impact of the current economic situation, with the COVID-19 pandemic having resulted in many enterprises going bankrupt; that is, it may not have been easy for college students to handle the negative impacts of entrepreneurship. Furthermore, the questionnaire data showed that most students had not experienced entrepreneurial failure and thus, could not positively judge the entrepreneurial experience. In addition, college students lack a certain degree of frustration education, and their acceptance of failure is limited.

Experiential learning and social network learning had a significant positive effect on entrepreneurial intention; however, there was no significant relationship between entrepreneurial education learning and entrepreneurial intention, a finding that goes against those in previous research, wherein entrepreneurial education had a direct effect on entrepreneurial intention (Crant, 1996; Souitaris et al., 2007; Anwar and Saleem, 2019; Jiatong et al., 2021; Aliedan et al., 2022). In our study, nonetheless, the path coefficient between the two is only 0.039, and this could have been, again, related to the COVID-19 epidemic. On this topic, Kuckertz (2013) showed that the direct impact of entrepreneurship education learning on entrepreneurial intention was primarily due to the practical learning style of entrepreneurship education. Still, to prevent the spread of COVID-19, colleges and universities across China adopted policies aimed at minimizing contact, such as implementing online teaching activities. This may have affected practical entrepreneurship education learning to a certain extent. Specifically, online teaching may have enabled only for theoretical entrepreneurship education to be carried out, which is bound to have an insufficient impact on entrepreneurship intention.

Our results also showed that entrepreneurial self-efficacy mediated the relationships of entrepreneurial education learning, experiential learning, social network learning and entrepreneurial intention, concurring with the findings of previous studies (Zhao et al., 2005; Wilson et al., 2007; Saeed et al., 2015; Li et al., 2020; Jiatong et al., 2021). This is because entrepreneurship education learning can enhance college students’ entrepreneurial self-awareness, social network learning can increase college students’ entrepreneurial self-confidence, and experiential learning can promote college students’ entrepreneurial practice experience. Thus, to enhance the entrepreneurial self-efficacy of college students, the entrepreneurial intention of college students must be improved.

### 7.2. Research conclusion

According to the above empirical conclusions, only H1 and H5 were not supported, albeit the two dimensions related to H5 showed a significant negative impact. Hereinafter we describe the theoretical and practical implications of the study.

Regarding theoretical implications, our study supplements the current evidence on the impact of various types of entrepreneurial learning on entrepreneurial intention, as well as newly introduced entrepreneurial self-efficacy as an intermediary variable to build a conceptual model comprising entrepreneurial learning, entrepreneurial self-efficiency, and entrepreneurial intention. The study also empirically tested the intermediary role of entrepreneurial self-efficacy on the relation of entrepreneurial learning and entrepreneurial intention, and clarified its internal relations. In sum, this research enriches entrepreneurial learning theory and provides the theoretical model of entrepreneurial learning-entrepreneurial self-efficacy-entrepreneurial intention.

Regarding practical implications, by putting forward the aforementioned theoretical model with entrepreneurial self-efficacy and testing it empirically, we help improve the theoretical framework of entrepreneurship and deliver related empirical evidence that may be useful for implementers and decision-makers of entrepreneurial education to develop effective learning programs, which may in turn enhance college students’ entrepreneurial intention. Based on the empirical results, government departments, universities, and relevant decision-making departments can continuously improve the social environment of entrepreneurial learning, effectively promote college students’ entrepreneurial self-efficacy, innovation ability, and entrepreneurial behavior. This may then help promote the healthy development of the whole social economy.

Our evidence allows us to put forward the following three suggestions. First, by strengthening the cooperation with enterprises, colleges and universities can: further promote the integration of industry and education and effectively combine theory and practice; stimulate college students’ entrepreneurial thinking and interest in class; create a good entrepreneurial atmosphere; strengthen the construction of an entrepreneurship education curriculum; enhance the practical training link in college students’ innovation and entrepreneurship curriculum; improve college students’ learning investment in entrepreneurship curriculum in order to promote in them a sense of entrepreneurial self-efficacy. Through different learning methods used entrepreneurship education, activities aimed at enhancing students’ sense of entrepreneurial self-efficacy could be consciously added to the design and implementation of curriculum teaching, so that college students can give positive and valuable feedback while learning, overcome their difficulties in entrepreneurship practice projects, and interaction quality can be improved.

Second, the government and universities can create a good social environment for, and improve the ecosystem of, entrepreneurship learning. Particularly, they can set up entrepreneurship competitions for college students, provide free places for the practice of entrepreneurship, and strengthen positive guidance and frustration education when facing college students’ experiential learning education. This can enable college students to improve their understanding of entrepreneurship, as well as accumulate experience and increase acceptance of entrepreneurship failure in advance.

Third, Chinese traditional Confucian culture pays more attention to the individual interests brought by Guanxi, which in turn deeply affects the potential entrepreneurs’ entrepreneurial learning. Chinese universities should, thus, give due attention to entrepreneurial learners’ social communication skills development, improve their information technology ability, and give them more opportunities to form social entrepreneurial networks with peer entrepreneurs to promote their entrepreneurial intention. In addition, schools and government agencies could, in addition to financial policy support, provide support for potential entrepreneurs by giving them opportunities to engage with entrepreneurship mentors and social entrepreneurship networks.

### 7.3. Research limitations and future prospects

While the current findings enrich entrepreneurial learning theory, this study is not without its limitations. First, this study only investigated students in Zhejiang Province, which limits generalizability to other provinces and cities in China. Due to China’s vast territory and abundant resources, different geographical conditions may entail variations in entrepreneurial environments and resources, which may influence entrepreneurial learning. Therefore, future studies should expand the sample size and region to improve research applicability.

Second, experiential learning had a significant negative impact on entrepreneurial self-efficacy, which conflicts with many previous findings. As this study did not comprehensively analyze the reasons for this discrepancy, future studies should focus on the specific contributing factors for this impact.

Third, this study explored the impacts of entrepreneurial learning, self-efficacy, and intention without controlling for profession, gender, and other variables. Future studies should further analyze the impacts of these factors on entrepreneurial intention to improve the accuracy of any results. In addition, under the background of Chinese traditional culture, it may also be a worthwhile endeavor for future researchers to explore the impact of Guanxi on college students’ entrepreneurial intention.

## Data availability statement

The original contributions presented in this study are included in the article/Supplementary material, further inquiries can be directed to the corresponding author.

## Ethics statement

Written informed consent was obtained from the individual(s)/minor(s)’ legal guardian/next of kin, for the publication of any potentially identifiable images or data included in this article.

## Author contributions

YP and YY wrote part of the first draft of the manuscript. LF contributed to the translation and documentation of the manuscript. ZC made several changes to different versions of the manuscript. CL made efforts to write the manuscript as a whole and empirical research. All the authors made corresponding contributions and agree to be accountable for the content of the work.

## References

[B1] Alam S. S. Mohd R. Kamarauddin B. H. Nor N. G. M. (2015). Personal values and entrepreneurial orientations in Malay entrepreneurs in Malaysia: Mediating role of self-efficacy. *Int. J. Commer. Manag.* 25 385–401. 10.1108/IJCoMA-01-2013-0001

[B2] Aldrich H. (1999). *Organizations evolving.* Thousand Oaks, CA: Sage.

[B3] Aldrich H. Zimmer C. (1985). “Entrepreneurship through social networks,” in *The art and science of entrepreneurship*, eds Sexton D. L. Smilor R. (Cambridge, MA: Ballinger), 3–47.

[B4] Aliedan M. M. Elshaer I. A. Alyahya M. A. Sobaih A. E. E. (2022). Influences of university education support on entrepreneurship orientation and entrepreneurship intention: Application of theory of planned behavior. *Sustainability* 14:13097. 10.3390/su142013097

[B5] Anselm M. (1993). “Entrepreneurship education in the community college,” in *Proceedings of the 38th international council for small business (ICSB)*, Las Vegas, NV, 177–192.

[B6] Anwar I. Saleem I. (2019). Exploring entrepreneurial characteristics among university students: An evidence from India. *Asia Pac. J. Innov. Entrepr.* 13 282–295. 10.1108/APJIE-07-2018-0044

[B7] Bagozzi R. P. Yi Y. (2012). Specifications, evaluation, and interpretation of structural equation models. *J. Acad. Mark. Sci.* 40 8–34. 10.1007/s11747-011-0278-x

[B8] Bandura A. (1977). Self-efficacy: Toward a unifying theory of behavioral change. *Psychol. Rev.* 84 191–215. 10.1037/0033-295X.84.2.191 847061

[B9] BarNir A. Watson W. E. Hutchins H. M. (2011). Mediation and moderated mediation in the relationship among role models, self-efficacy, entrepreneurial career intention, and gender. *J. Appl. Soc. Psychol.* 41 270–297. 10.1111/j.1559-1816.2010.00713.x

[B10] Baughn C. C. Cao J. S. R. Le L. T. M. Lim V. A. Neupert K. E. (2006). Normative, social and cognitive predictors of entrepreneurial interest in China, Vietnam and the Philippines. *J. Dev. Entrep.* 11 57–77. 10.1142/S108494670600026X

[B11] Bird B. (1988). Implementing entrepreneurial ideas: The case for intention. *Acad. Manag. Rev.* 13 442–453. 10.2307/258091

[B12] Boyd N. G. Vozikis G. S. (1994). The influence of self-efficacy on the development of entrepreneurial intentions and actions. *Entrep. Theory Pract.* 18 63–77. 10.1177/104225879401800404

[B13] Bullough A. Renko M. Myatt T. (2014). Danger zone entrepreneurs: The importance of resilience and self-efficacy for entrepreneurial intentions. *Entrep. Theory Pract.* 38 473–499. 10.1111/etap.12006

[B14] Chen C. C. Greene P. G. Crick A. (1998). Does entrepreneurial self-efficacy distinguish entrepreneurs from managers? *J. Bus. Ventur.* 13 295–316. 10.1016/S0883-9026(97)00029-3

[B15] Chien-Chi C. Sun B. Yang H. Zheng M. Li B. (2020). Emotional competence, entrepreneurial self-efficacy, and entrepreneurial intention: A study based on China college students’ social entrepreneurship project. *Front. Psychol.* 11:547627. 10.3389/fpsyg.2020.547627 33312146 PMC7704431

[B16] Chou C. M. Shen C. H. Hsiao H. C. (2011). The influence of entrepreneurial self-efficacy on entrepreneurial learning behavior-using entrepreneurial intention as the mediator variable. *Int. Bus. Manag.* 3 7–11.

[B17] Cope J. (2005). Toward a dynamic learning perspective of entrepreneurship. *Entrep. Theory Pract.* 29 373–397. 10.1111/j.1540-6520.2005.00090.x

[B18] Corbett A. C. (2007). Learning asymmetries and the discovery of entrepreneurial opportunities. *J. Bus. Ventur.* 22 97–118. 10.1016/j.jbusvent.2005.10.001

[B19] Crant J. M. (1996). The proactive personality scale as a predictor of entrepreneurial intentions. *J. Small Bus. Manag.* 34 42–50.

[B20] Davidsson P. Honig B. (2003). The role of social and human capital among nascent entrepreneurs. *J. Bus. Ventur.* 18 301–331. 10.1016/S0883-9026(02)00097-6

[B21] De Noble A. F. Jung D. Ehrlich S. B. (1999). “Entrepreneurial self-efficacy: The development of a measure and its relationship to entrepreneurial actions,” in *Frontiers of entrepreneurship research*, eds Reynolds P. D. Bygrave W. D. Manigar S. Mason C. M. Meyer G. D. Sapienza H. J. (Waltham, MA: P. R. Publication), 73–87.

[B22] Dehghanpour Farashah A. (2013). The process of impact of entrepreneurship education and training on entrepreneurship perception and intention: Study of educational system of Iran. *Educ. Train.* 55 868–885. 10.1108/ET-04-2013-0053

[B23] Drucker P. F. (1985). *Innovation and entrepreneurship: Practice and principles.* New York, NY: Harper & Row.

[B24] Fayolle A. Gailly B. Lassas-Clerc N. (2006). Assessing the impact of entrepreneurship education programmes: A new methodology. *J. Eur. Ind. Train.* 30 701–720. 10.1108/03090590610715022

[B25] Florin J. Karri R. Rossiter N. (2007). Fostering entrepreneurial drive in business education: An attitudinal approach. *J. Manag. Educ.* 31:17242. 10.1177/1052562905282023

[B26] Fornell C. Larcker D. F. (1981). Evaluating structural equation models with unobservable variables and measurement error. *J. Market. Res.* 18 39–50. 10.2307/3151312

[B27] Fragoso R. Rocha-Junior W. Xavier A. (2020). Determinant factors of entrepreneurial intention among university students in Brazil and Portugal. *J. Small Bus. Entrep.* 32 33–57. 10.1080/08276331.2018.1551459

[B28] Franke N. Lüthje C. (2004). Entrepreneurial intentions of business students: A benchmarking study. *Int. J. Innov. Technol. Manag.* 1 269–288. 10.1142/S0219877004000209

[B29] French A. M. Luo X. R. Bose R. (2017). Toward a holistic understanding of continued use of social networking tourism: A mixed-methods approach. *Inf. Manag.* 54 802–813. 10.1016/j.im.2016.10.006

[B30] Gwynne P. (2008). More schools teaching entrepreneurship. *Res. Technol. Manag.* 51 6–8.

[B31] Hansen E. L. (1995). Entrepreneurial networks and new organization growth. *Entrep. Theory Pract.* 19 7–19. 10.1177/104225879501900402

[B32] Herron L. Sapienza H. J. (1992). The entrepreneur and the initiation of new venture launch activities. *Entrep. Theory Pract.* 17 49–55. 10.1177/104225879201700106

[B33] Hills G. E. Lumpkin G. T. Singh R. P. (1997). “Opportunity recognition: Perceptions and behaviors of entrepreneurs,” in *Frontiers of entrepreneurship research*, ed. Reynolds P. D. (Babson Park, FL: Babson College), 203–218.

[B34] Holcomb T. R. Ireland R. D. Holmes R. M. Hitt M. A. (2009). Architecture of entrepreneurial learning: Exploring the link among heuristics, knowledge, and action. *Entrep. Theory Pract.* 33 167–192. 10.1111/j.1540-6520.2008.00285.x

[B35] Jackson D. L. Gillaspy J. A. Purc-Stephenson R. (2009). Reporting practices in confirmatory factor analysis: An overview and some recommendations. *Psychol. Methods* 14 6–23. 10.1037/a0014694 19271845

[B36] Jiang H. Xiong W. Cao Y. (2017). Research on the mechanism of entrepreneurial education quality, entrepreneurial self-efficacy and entrepreneurial intention in social sciences, engineering and science education. *EURASIA J. Math. Sci. Technol. Educ.* 13 3709–3721. 10.12973/eurasia.2017.00754a

[B37] Jiatong W. Murad M. Bajun F. Tufail M. S. Mirza F. Rafiq M. (2021). Impact of entrepreneurial education, mindset, and creativity on entrepreneurial intention: Mediating role of entrepreneurial self-efficacy. *Front. Psychol.* 12:724440. 10.3389/fpsyg.2021.724440 34497568 PMC8419428

[B38] Katz J. A. (1992). Psychological cognitive model of employment status choice. *Entrep. Theory Pract.* 17 29–37. 10.1177/104225879201700104

[B39] Kim J. Miner A. S. (2007). Vicarious learning from the failure and near-failure of others: Evidence from the U.S. commercial banking industry. *Acad. Manag. J.* 50 687–714. 10.5465/amj.2007.25529755

[B40] Kirzner I. M. (1973). *Competition and entrepreneurship.* Chicago, IL: University of Chicago Press.

[B41] Kolb D. A. (1984). *Experiential learning: Experience as the source of learning and development.* Englewood Cliffs, NJ: Prentice-Hall.

[B42] Krueger N. F. Carsrud A. L. (1993). Entrepreneurial intentions: Applying the theory of planned behavior. *Entrep. Reg. Dev.* 5 315–330. 10.1080/08985629300000020

[B43] Krueger N. F. Reilly M. Carsrud A. L. (2000). Competing models of entrepreneurial intentions. *J. Bus. Ventur.* 15 411–432. 10.1016/S0883-9026(98)00033-0

[B44] Kuckertz A. (2013). Entrepreneurship education–status quo and prospective developments. *J. Entrep. Educ.* 16 59–71. 10.2139/ssrn.1862295

[B45] Li H. Wang J. Zhang Y. Li H. Chen X. (2020). The impact of self-efficacy analysis-based psychological theory and literary ethics on Chinese American entrepreneurship education. *Front. Psychol.* 11:1870. 10.3389/fpsyg.2020.01870 32849097 PMC7417519

[B46] Liñán F. Chen Y. W. (2009). Development and cross-cultural application of a specific instrument to measure entrepreneurial intentions. *Entrep. Theory Pract.* 33 593–617. 10.1111/j.1540-6520.2009.00318.x

[B47] Liñán F. Rodríguez-Cohard J. C. Rueda-Cantuche J. M. (2005). Factors affecting entrepreneurial intention levels: A role for education. *Int. Entrep. Manag. J.* 7 195–218. 10.1007/s11365-010-0154-z

[B48] Liu X. Lin C. Zhao G. Zhao D. (2019). Research on the effects of entrepreneurial education and entrepreneurial self-efficacy on college students’ entrepreneurial intention. *Front. Psychol* 10:869. 10.3389/fpsyg.2019.00869 31068862 PMC6491517

[B49] Lombard M. Snyder-Duch J. Bracken C. C. (2002). Content analysis in mass communication: Assessment and reporting of intercoder reliability. *Hum. Commun. Res.* 28 587–604. 10.1111/j.1468-2958.2002.tb00826.x

[B50] Lumpkin G. T. Lichtenstein B. B. (2005). The role of organizational learning in the opportunity-recognition process. *Entrep. Theory Pract.* 29 451–472. 10.1111/j.1540-6520.2005.00093.x

[B51] Luo Y. F. Huang J. Gao S. (2022). Relationship between proactive personality and entrepreneurial intentions in college students: Mediation effects of social capital and human capital. *Front. Psychol.* 13:861447. 10.3389/fpsyg.2022.861447 35783804 PMC9243360

[B52] Lv Y. Chen Y. Sha Y. Wang J. An L. Chen T. (2021). How entrepreneurship education at universities influences entrepreneurial intention: Mediating effect based on entrepreneurial competence. *Front. Psychol.* 12:655868. 10.3389/fpsyg.2021.655868 34295281 PMC8289882

[B53] Mason C. Arshed N. (2013). Teaching entrepreneurship to university students through experiential learning: A case study. *Ind. High. Educ.* 27 449–463. 10.5367/ihe.2013.0180

[B54] McGee J. E. Peterson M. (2019). The long-term impact of entrepreneurial self-efficacy and entrepreneurial orientation on venture performance. *J. Small Bus. Manag.* 57 720–737. 10.1111/jsbm.12324

[B55] Minniti M. Bygrave W. (2001). A dynamic model of entrepreneurial learning. *Entrep. Theory Pract.* 25 5–16. 10.1177/104225870102500301

[B56] Ni N. Wang Z. (2005). Reflection on entrepreneurial learning. *Sci. Res. Manag.* 26 95–96.

[B57] Noy C. (2008). Sampling knowledge: The hermeneutics of snowball sampling in qualitative research. *Int. J. Soc. Res. Methodol.* 11 327–344. 10.1080/13645570701401305

[B58] Nunkoo R. Ramkissoon H. Gursoy D. (2013). Use of structural equation modeling in tourism research: Past, present, and future. *J. Travel Res.* 52 759–771. 10.1177/0047287513478503

[B59] Obschonka M. Hakkarainen K. Lonka K. Salmela-Aro K. (2017). Entrepreneurship as a twenty-first century skill: Entrepreneurial alertness and intention in the transition to adulthood. *Small Bus. Econ.* 48 487–501. 10.1007/s11187-016-9798-6

[B60] Petkova A. P. (2009). A theory of entrepreneurial learning from performance errors. *Int. Entrep. Manag. J.* 5:345. 10.1007/s11365-008-0075-2

[B61] Politis D. (2005). The process of entrepreneurial learning: A conceptual framework. *Entrep. Theory Pract.* 29 399–424. 10.1111/j.1540-6520.2005.00091.x

[B62] Rae D. Carswell M. (2000). Using a life-story approach in researching entrepreneurial learning: The development of a conceptual model and its implications in the design of learning experiences. *Educ. Train.* 42 220–227. 10.1108/00400910010373660

[B63] Rae D. Carswell M. (2001). Towards a conceptual understanding of entrepreneurial learning. *J. Small Bus. Enterp. Dev.* 8 150–158. 10.1108/EUM0000000006816

[B64] Ramsgaard M. B. Østergaard S. J. (2018). An entrepreneurial learning approach to assessment of internships. *Educ. Train.* 60 909–922. 10.1108/ET-11-2016-0164

[B65] Roy R. Akhtar F. Das N. (2017). Entrepreneurial intention among science & technology students in India: Extending the theory of planned behavior. *Int. Entrep. Manag. J.* 13 1013–1041. 10.1007/s11365-017-0434-y

[B66] Saeed S. Yousafzai S. Y. Yani-De-Soriano M. Muffatto M. (2015). The role of perceived university support in the formation of students’ entrepreneurial intention. *J. Small Bus. Manag.* 53 1127–1145. 10.1111/jsbm.12090

[B67] Scherer R. F. Adams J. S. Carley S. S. Wiebe F. A. (1989). Role model performance effects on development of entrepreneurial career preference. *Entrep. Theory Pract.* 13 53–72. 10.1177/104225878901300306

[B68] Schumpeter J. A. (1934). *The theory of economic development: An inquiry into profits, capital, credit, interest and the business cycle.* Cambridge, MA: Harvard.

[B69] Sequeira J. Mueller S. L. McGee J. (2007). The influence of social ties and self-efficacy in forming entrepreneurial intentions and motivating nascent behavior. *J. Dev. Entrep.* 12 275–293. 10.1142/S108494670700068X

[B70] Souitaris V. Zerbinati S. Al-Laham A. (2007). Do entrepreneurship programmes raise entrepreneurial intention of science and engineering students? The effect of learning, inspiration and resources. *J. Bus. Ventur.* 22 566–591. 10.1016/j.jbusvent.2006.05.002

[B71] Thompson E. R. (2009). Individual entrepreneurial intent: Construct clarification and development of an internationally reliable metric. *Entrep. Theory Pract.* 33 669–694. 10.1111/j.1540-6520.2009.00321.x

[B72] Tsui A. S. Farh J. L. (1997). Where Guanxi matters: Relational demography and Guanxi in the Chinese context. *Work Occup.* 24 56–79. 10.1177/0730888497024001005

[B73] van Gelderen M. Brand M. van Praag M. Bodewes W. Poutsma E. van Gils A. (2008). Explaining entrepreneurial intentions by means of the theory of planned behaviour. *Career Dev. Int.* 13 538–559. 10.1108/13620430810901688

[B74] Weick K. E. (1995). *Sensemaking in organizations.* Thousand Oaks, CA: Sage Publications.

[B75] Wilson F. Kickul J. Marlino D. (2007). Gender, entrepreneurial self-efficacy, and entrepreneurial career intentions: Implications for entrepreneurship education. *Entrep. Theory Pract.* 31 387–406. 10.1111/j.1540-6520.2007.00179.x

[B76] Wood R. Bandura A. (1989). Social cognitive theory of organizational management. *Acad. Manag. Rev.* 14 361–384. 10.5465/amr.1989.4279067

[B77] Wu L. Jiang S. Wang X. Yu L. Wang Y. Pan H. (2021). Entrepreneurship education and entrepreneurial intentions of college students: The mediating role of entrepreneurial self-efficacy and the moderating role of entrepreneurial competition experience. *Front. Psychol.* 12:727826. 10.3389/fpsyg.2021.727826 35069312 PMC8770817

[B78] Yang P. Wang Q. Jing M. (2021). Study on the influence of learning engagement on entrepreneurial intention of college students. *J. Natl Inst. Educ. Admin.* 1:85–95.

[B79] Zhang Y. Duysters G. Cloodt M. (2014). The role of entrepreneurship education as a predictor of university students’ entrepreneurial intention. *Int. Entrep. Manag. J.* 10 623–641. 10.1007/s11365-012-0246-z

[B80] Zhao H. Seibert S. E. Hills G. E. (2005). The mediating role of self-efficacy in the development of entrepreneurial intentions. *J. Appl. Psychol.* 90 1265–1272. 10.1037/0021-9010.90.6.1265 16316279

