# Supplementary material for: The influence mechanism of the relationship between entrepreneurial learning and entrepreneurial intention
Source: Front Psychol. 2023 Jan 18;13:1023808. doi: 10.3389/fpsyg.2022.1023808 (PMC9891665; doi:10.3389/fpsyg.2022.1023808)
Supplement: Supplementary file 1 [file Table_1.DOCX]

Supplementary Material

The Influence Mechanism of the Relationship Between Entrepreneurial Learning and Entrepreneurial Intention

**Cong Lin^*^, Yan Pan, Yanli Yu, Libo Feng, Zhiyong Chen**

*** Correspondence:** Corresponding Author：[24414537@qq.com](mailto:24414537@qq.com)

**Supplementary Tables**

Supplementary Table 1. Study 1 participants’ background information (N=15)

| **Name** | **Age** | **Gender** | **Education** | **Major** | **Place of work** | **Industry** |
| --- | --- | --- | --- | --- | --- | --- |
| Wang | 29 | Male | Master | Applied chemistry | Hangzhou | Manufacturing |
| Zhao | 28 | Male | Undergraduate | International trade | Hangzhou | Transportation industry |
| Zhang | 22 | Male | Undergraduate | Logistics management | Hangzhou | Electronic commerce |
| Lin | 28 | Female | Undergraduate | Law | Ningbo | Business services |
| Li | 25 | Male | Undergraduate | Environmental science engineering | Ningbo | Manufacturing |
| Wu | 24 | Male | Undergraduate | Tourism management | Hangzhou | Catering industry |
| Chen | 27 | Male | Undergraduate | Electrical automation | Ningbo | Technical service industry |
| Xu | 23 | Male | Undergraduate | Mechanical and electronic engineering | Wenzhou | Manufacturing |
| Sun | 27 | Male | Undergraduate | International trade | Wenzhou | Foreign trade |
| Wang | 30 | Male | Master | Economics | Wenzhou | Electronic commerce |
| Ni | 26 | Male | Undergraduate | Communication engineering | Hangzhou | Computer services |
| Zhang | 27 | Female | Undergraduate | Computer science and technology | Hangzhou | Healthcare industry |
| Ma | 23 | Male | Undergraduate | Chinese language and literature | Ningbo | Education and training |
| Zhu | 25 | Male | Undergraduate | Business administration | Ningbo | Electronic commerce |
| Yang | 27 | Female | Undergraduate | Accounting | Hangzhou | Electronic commerce |
